# A compact acoustic spanner to rotate macroscopic objects

**DOI:** 10.1038/s41598-019-43046-4

**Published:** 2019-05-01

**Authors:** Ermes Toninelli, Mitchell A. Cox, Graham M. Gibson, Stuart D. Brown, Matthew P. Edgar, Andrew Forbes, Miles J. Padgett

**Affiliations:** 1 SUPA, School of Physics and Astronomy, University of Glasgow, Glasgow, G12 8QQ UK; 2 School of Electrical and Information Engineering, University of the Witwatersrand, Johannesburg, ZA South Africa; 3 School of Physics, University of the Witwatersrand, Johannesburg, ZA South Africa

**Keywords:** Electrical and electronic engineering, Acoustics

## Abstract

Waves can carry both linear and angular momentum. When the wave is transverse (e.g. light), the angular momentum can be characterised by the “spin” angular momentum associated with circular polarisation, and the “orbital” angular momentum (OAM) arising from the phase cross-section of the beam. When the wave is longitudinal (e.g. sound) there is no polarization and hence no spin angular momentum. However, a suitably phase-structured sound beam can still carry OAM. Observing the transfer of OAM from sound to a macroscopic object provides an excellent opportunity to study the exchange of energy between waves and matter. In this paper we show how to build a compact free-space acoustic spanner based on a 3D-printed sound-guiding structure and common electronic components. We first characterise the sound fields by measuring both phase and amplitude maps, and then show a video of our free-space acoustic spanner in action, in which macroscopic objects spin in a circular motion and change direction of rotation according to the handedness of the OAM acoustic field.

## Introduction

That light carries a linear momentum in addition to its energy has been recognised at least since the time of Kepler who hypothesized that the tails of comets always pointed away from the sun due to the optical radiation pressure exerted by the solar wind^1^. It is easy to show that the corresponding force exerted by a light beam is equal to the power transmitted by the beam divided by the phase velocity of the wave, meaning that a typical laser pointer exerts a few pico Newtons upon a screen, due to the very large value of the speed of light^2^. This relationship between power and phase velocity covers not just light, but all wave types, including sound waves. It follows that sound propagating in air exerts a force a million times higher than an optical beam of the same power, owing to the comparatively much slower speed of sound.

In addition to linear momentum, waves can also carry angular momentum. When the wave is transverse this angular momentum can be characterised by the “spin” angular momentum, associated with circular polarisation, nicely described by a mechanical analogy by Poynting^3^ and demonstrated by Beth^4^. In addition to this spin angular momentum, waves can also carry OAM associated with the phase cross-section of the wave. This OAM was recognised by Darwin^5^ in the 1930’s but it was not until the work of Allen and co-workers in the 1990s that OAM was specifically linked to beams of light with helical phase-fronts^6^. The transfer of linear and both spin and orbital angular momentum to the motion of microscopic particles has been beautifully demonstrated in optical tweezers and optical spanners respectively^7–9^. While a longitudinal sound wave has no polarisation and hence cannot carry spin angular momentum, it can however carry OAM when imparted with an appropriate azimuthal phase structure. A helical wave with a transverse azimuthal phase dependence of ℓ*ϕ* carries an OAM with an OAM to power ratio of ℓ*ω*, where ℓ is the topological charge of the singularity and can be any integer value positive or negative, *ϕ* is the phase, and *ω* is the frequency of the wave.

Many research groups world-wide have studied the transfer of optical momentum but comparatively fewer have looked at the acoustic transfer. First to show the transfer of an acoustic OAM was Volke-Sepiúlveda and co-workers^10^, and subsequent work by others^11–14^ having demonstrated various acoustic analogies to optical angular momentum effects. In most of this previous work the helically-phased acoustic beam has been created using a ring of loudspeakers, each driven at the same angular frequency *ω*_*s*_, but with an azimuthally changing phase delay given by $\ell \cdot \theta$.

**Figure 1:**
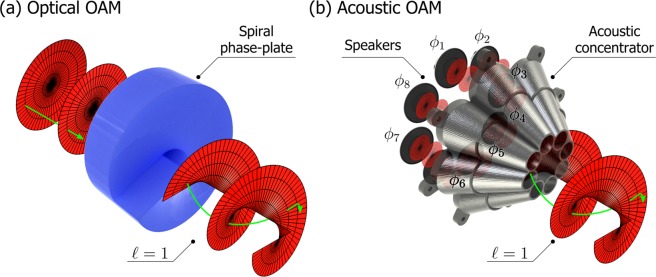
Generation of optical and acoustic OAM. Optical OAM can be imparted on incoming plane waves by a spiral phase-plate: an optical element with an azimuthally variable phase. In the case of acoustic OAM, a phased-array of speakers (${\varphi }_{1{\rm{to8}}}(\ell =\mathrm{1)}=0\,{\rm{to}}\,2\pi$) can be used to generate the structured acoustic field. We use an acoustic concentrator of our design to focus the field. In the presence of OAM, the Poynting vector (green arrow) has an azimuthal component and is no longer parallel to the beam axis.

The physical origin of angular momentum and its subsequent transfer to matter is on the one hand mathematically complicated but on the other conceptually simple. Whereas optical OAM can be generated using a spiral phase-plate, as shown in Fig. 1(a), or a more modern computer-generated ‘forked’ hologram displayed on a spatial light modulator, acoustic OAM can be generated using a phased-array of speakers, as shown in Fig. 1(b). In the case of light it is also possible to generate OAM beams and more generally vector beams by the use of spin-orbit coupling devices, which can either be liquid-crystal based or consist of wavefront-shaping metasurfaces (see the recent review articles by Rosales-Guzmán *et al*.^15^ and Ambrosio^16^ and references therein, for a comprehensive discussion about vector light fields and light-structuring metasurfaces).

The physical origin of the OAM is easy to understand. As recognised by Allen *et al*. OAM arises from helical phase-fronts. At each position in a helically-phased beam the local energy and momentum flow, as represented by the Poynting and k-vector respectively, are tilted with respect to the beam-axis. For a helical phase described by $\exp (i\ell \varphi )$, simple geometry shows this tilt angle to be $\ell \lambda /2\pi r$^17^. Because of this tilt, at all positions in the beam, the linear momentum has a component in the *ϕ*-direction which for a single photon is given as $\hslash {k}_{0}\ell \lambda /2\pi r$, i.e the key result of Allen *et al*. Expressed in ratio terms, the orbital angular momentum to energy ratio is ℓ/*ω*, for a helically-phased wave. Having considered the origins of the OAM for helically-phased light, it is widely recognised that a similar argument for all waves, and acoustic waves in particular. It remains the case that the helical phase-fronts mean that the momentum in the wave is tilted with respect to the beam-axis, again giving a *ϕ*-component and, when summed, an angular momentum about the beam-axis. For fuller discussion the reader is directed to the work of Hefner and Marston^18^, and G. Spalding and co-workers^19,20^. Previously, spatially complex sound-fields have been generated in the ultrasonic regime using large arrays of piezoelectric transducers^21–23^. Moreover, the metasurface approach can also be applied to the creation of acoustic OAM^24,25^.

In this work we show how to build a compact acoustic spanner capable of transferring angular momentum to macroscopic objects, causing them to rotate. The spanner is realised using a 3D-printed ‘funnel’-shaped acoustic concentrator and 8 mini-speakers driven by an Arduino microcontroller. In terms of both construction cost and complexity our open-source acoustic spanner can be built and further developed using similar rapid-prototyping techniques, with a view to demonstrating the transfer of wave momentum to matter.

## Results

Firstly, the ability of the acoustic spanner to produce OAM is verified by recording the intensity and phase maps of the acoustic fields associated to ℓ = 0, 1, and −1. Secondly, we show how a small object such as a polystyrene packing peanut (mass ≈ 3 g; dimensions ≈40 × ≈30 mm) placed in a Petri-dish can be set into rotation, either clock-wise or counter-clock-wise, by changing the handedness of the acoustic OAM field. Lastly, we show a qualitative representation of the acoustic OAM mode, by computing the optical flow of an ensemble of graphite-flakes floating on the surface of water. Videos of the acoustic spanner in action can be found in the Supplementary Information.

**Figure 2:**
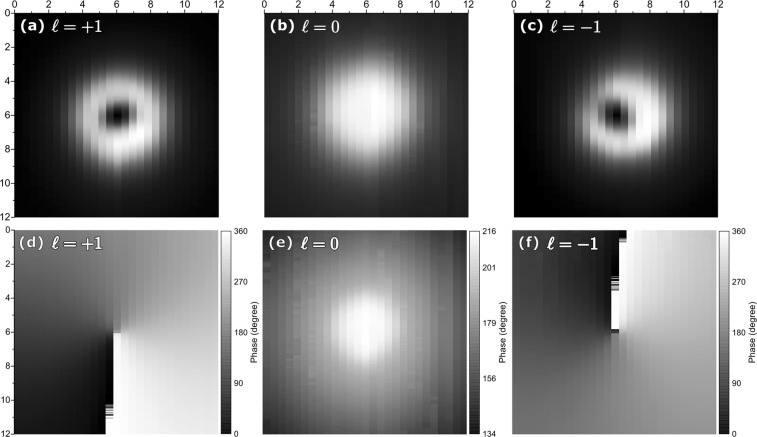
Intensity and phase maps of the acoustic fields generated by the 3D-printed acoustic spanner. (**a**–**c**) show the intensity maps for ℓ = +1, 0, 1, −1. The corresponding phase-maps are shown in (**d**–**f**). The x- and y- axes are measured in centimetres. Each vertical scan was taken at ≈0.5 cm intervals.

**Figure 3:**
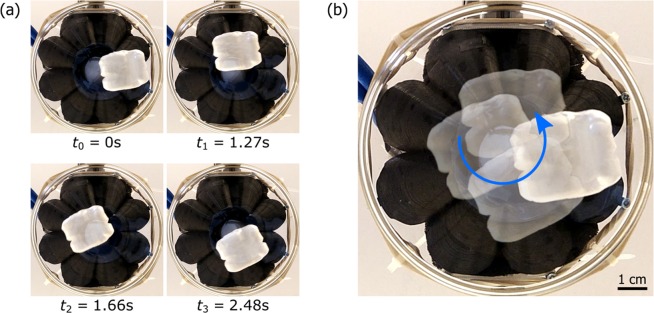
Rotational motion of macroscopic objects. A packing peanut is set into rotation by the intercepted OAM acoustic field. Friction causes uneven motion, as revealed by the time labels in (**a**). The overall motion of the rotating packing pellet is represented in (**b**). The slight asymmetry about the centre-point in the motion of the pellet confirms the uneven acoustic intensity shown in Fig. 2(c).

**Figure 4:**
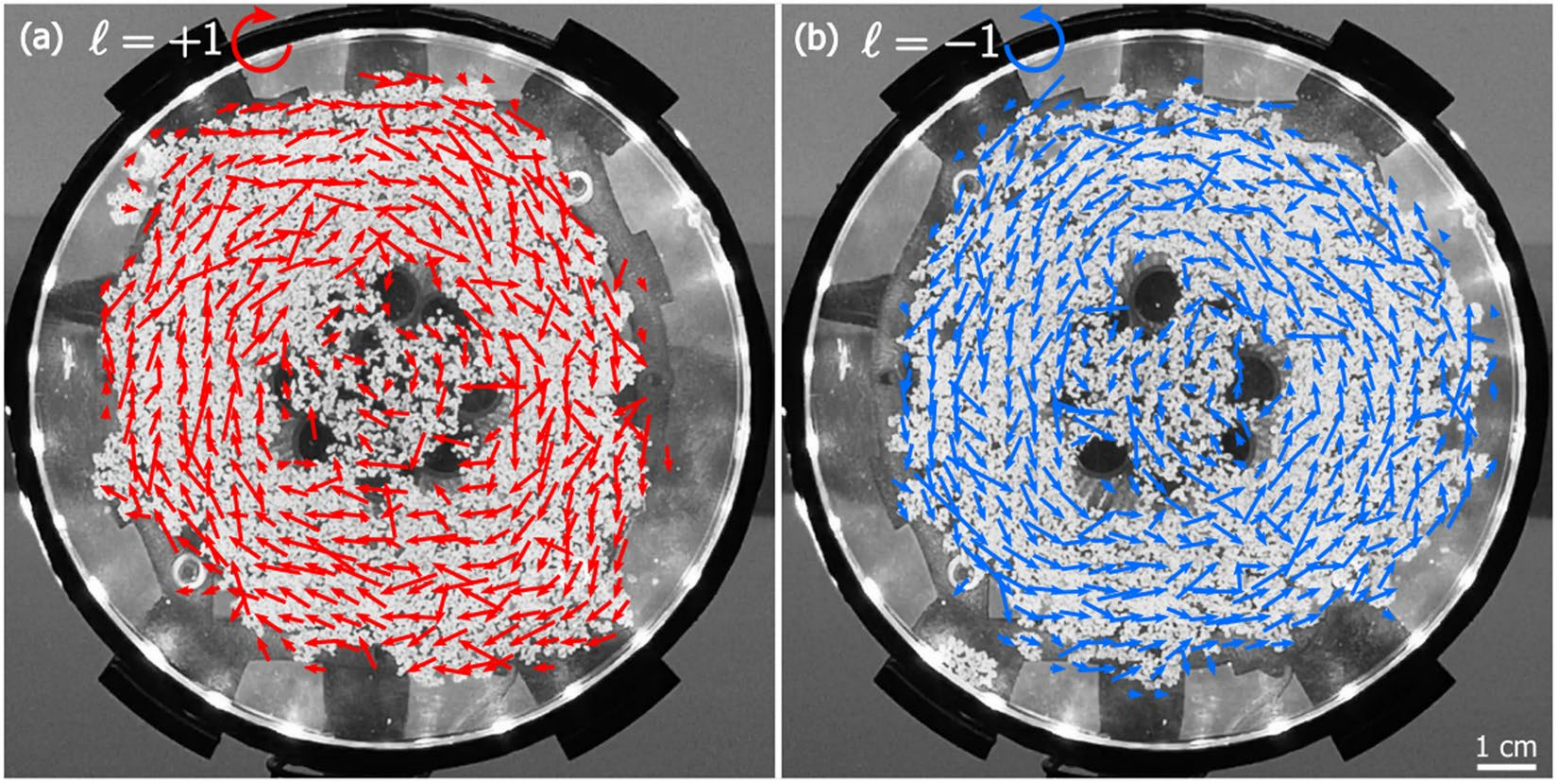
Qualitative mapping of the OAM acoustic field by optical-flow analysis of rotating particles. The circular motion of graphite flakes floating on the surface of water qualitatively reveals the generated acoustic OAM fields. The red/blue arrows highlight a counter-/clock-wise motion of the graphite flakes, which correspond to ℓ = +1 and ℓ = −1 respectively. The arrows point toward the direction of motion of the particles, and their length is is proportional to the speed of the particles. The slight asymmetry about the centre-point in the motion of the graphite flakes (most noticeable for ℓ = −1, as shown in (**b**)) confirms the uneven acoustic intensity shown in Fig. 2(c). The size-bar at the bottom-right applies to both images.

The intensity and phase acoustic-maps for ℓ = +1, 0, and −1 are shown in Fig. 2(a–f) respectively. The intensity-maps reveal the characteristic ‘donought’ shape of OAM beams for ℓ = ±1 (Fig. 2(a,c)). The intensity of the Gaussian acoustic-field for ℓ = 0 is also confirmed (Fig. 2(b)). The phase maps further confirm the accurate production of the desired acoustic-fields. It should be noted that the power output of the phased-array of speakers appears to be slightly uneven in the case of ℓ = −1, as confirmed in Fig. 2(c) and in the slight asymmetry visible in Figs 3(b) and 4(b). Further details on how the maps of the acoustic-fields were recorded is provided in the methods’ section.

Having characterised the acoustic fields, we proceeded to record videos of the acoustic spanner in action. In the first reported video, a packing-peanut can be seen rotating several cycles in each direction, as the handedness of the acoustic OAM is switched, from ℓ = +1 to ℓ = −1. When all of the speakers are driven in phase with each other, i.e. ℓ = 0, motion stops, and the packing-peanut remains stationary and unperturbed. The uneven shape of the packing-peanut and friction against the glass Petri dish means that at times its motion changes speed. A number of frames of the rotating-peanut are reported in Fig. 3(a), and the overall motion is represented in Fig. 3(b). We found that large, light objects are more easily set into motion, due to their larger cross-sectional area, which allows for a greater interaction with the acoustic field.

In the second reported video, the generated OAM field is qualitatively visualised by optical-flow analysis of the circular motion of several hundred particles (graphite flakes of ≈2 mm diameter), floating on the surface of water. Accordingly, the LabVIEW implementation of the Horn-Schunck optical-flow analysis was used to produce Fig. 4, for ℓ = +1 and ℓ = −1. More details about the optical-flow algorithm can be found in the work by B. Horn and B. Schunck^26^. This qualitative analysis confirms the general features of the acoustic OAM field: (1) the central region of the field is a mixture of phases which results in zero-intensity and therefore very limited circular motion of particles, as shown by the shorter length of the arrows (some motion is still present due to the flow of water upon which the particles float); (2) the annular region of the field is associated with fast motion of particles, as shown by the longer length of the arrows. In spite of the qualitative nature of this analysis, the ability to macroscopically visualise the acoustic field may enable an intuitive grasp of OAM and benefit our understanding of the underlying physics.

## Discussion

Whereas traditionally the generation of acoustic OAM required relatively complex apparatus to reliably generate audio signals with appropriate phase-differences, in our current work we share the designs for a free-space 3D-printed acoustic spanner that it is both compact and inexpensive. Ambitiously, our demonstration of a free-space actuation of macroscopic objects using acoustic OAM may aid the realisation of new laboratory techniques, in which the ability to rotate objects using sound-waves (as opposed to ultrasonic standing-waves) is desired.

## Methods

In this section we discuss the rapid prototyping techniques used to create the acoustic spanner. 3D-printing is used to construct the funnel-shaped acoustic concentrator, as well as the support structures required to link all of the components in place. The acoustic OAM field is generated via Direct Digital Synthesis (DDS) using the open hardware Arduino microcontroller platform, in combination with some custom electronics.

### 3D-printing

The first automatic method for fabricating a three-dimensional plastic model was demonstrated almost 40 years ago^27^. Since then, a huge proliferation of 3D-printing has made this game changing technology accessible to the layman, to the extent that 3D-printers can now replicate themselves, thanks to a symbiotic relationship with the user^28^. Following drastic improvements in material science and engineering, 3D-printed parts fabricated with inexpensive printers have proven to be suitable for laboratory use^29,30^. A few examples of ingenious 3D-printed based systems include the smart-phone laser beam spatial profiler^31^, the *μ*Cube-optomechanics framework^32^, and the one-piece flexure translation stage microscope^33^. Encouraged by the success of these and other projects, we decided to produce the first 3D-printed acoustic spanner, selecting what we believe to be the most accessible and powerful tools to both design the 3D-printed parts and synthesise the acoustic-OAM field. Accordingly, Autodesk Fusion 360 was used to design the funnel-shaped acoustic concentrator and the supporting structures. The designed parts were then printed using an Ultimaker 2 3D-printer, loaded with Polylactic acid (PLA) filament. A fully assembled acoustic spanner was then produced by simply fitting the speakers and linking the electronic boards.

**Figure 5:**
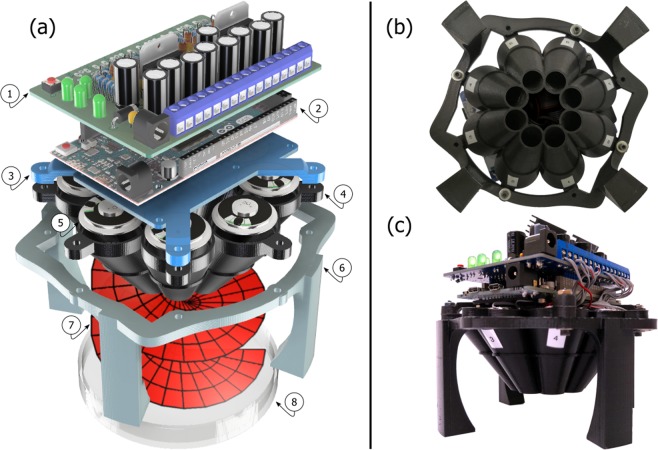
3D printed acoustic spanner. An exploded view of the main components of the acoustic spanner is shown in (**a**). These are: a custom built amplifiers and filters electronic board (1); an Arduino Due microcontroller (2); 3D-printed connecting structures (3) and (6); the 3D-printed ‘funnel’ acoustic-guide (4); eight mini-speakers (5); and a sample holder (8). The generated OAM acoustic field is artistically represented in (7). Photographs of the assembled acoustic spanner are shown from the bottom (**b**) and from the side (**c**).

An exploded view of the components of the acoustic spanner is shown in Fig. 5(a). The fully-assembled 3D-printed acoustic spanner is shown in Fig. 5(b,c). The design of the acoustic spanner had to satisfy the constraints introduced by the physical size of the components for a compact and inexpensive unit, while ensuring maximum transfer of sound-pressure to matter. The type and size of the loudspeakers was a determining factor. We opted for eight 28 mm diameter minispeakers (Visaton K28WP), which are both very compact and high-quality, thanks to their Mylar diaphragm and considerable rated power for their size (1W average power). The speakers were distributed around a 10 cm diameter circle. In order to concentrate the sound-field to an extent that macroscopic objects could be set into motion, a funnel-shaped acoustic concentrator was designed. This consisted of eight individual sound-guides, oriented at a skew angle. Accordingly, the diameter over which the sound of the minispeakers is produced was reduced from the original size of 10 cm to the 3 cm diameter of the small-end of the funnel. The angle at which the individual sound-guides are oriented was chosen so as to minimise the decay of the sound intensity with distance, while avoiding a too shallow angle which would effectively reflect most of the sound-field back to the speakers. Lastly, we took care in avoiding critically overhung structures, ensuring compatibility of our designed parts with most commonly available single filament 3D-printers, which may not be able to print adequate support structures.

### Acoustic OAM generation

**Figure 6:**
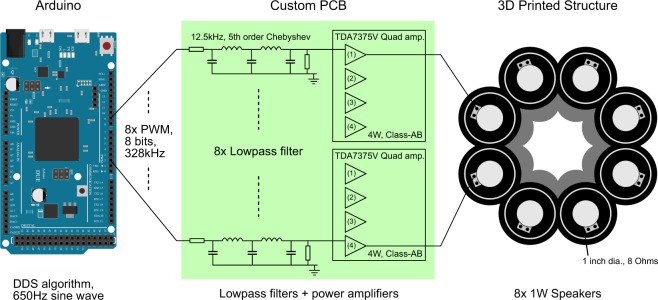
Block diagram of the Acoustic Spanner electronics. The DDS algorithms and PWM signals are implemented on an Arduino Due microcontroller platform. A custom PCB contains the individual low-pass filters and power amplifiers for generating the audio signals. The amplified audio signals are fed to their corresponding loudspeakers, which are housed in a 3D printed funnel-shaped acoustic-guide structure. Some connections to the loudspeakers have been omitted for clarity.

In this section we discuss our approach to the generation of 8 sinusoidal audio-signals with determined initial phases, required to drive the phased-array of speakers and produce acoustic OAM. The Acoustic Spanner utilizes a phased array of eight loudspeakers driven at 650 Hz. This frequency was chosen based on the frequency response of the loudspeakers enabling maximum conversion efficiency of electrical power into acoustic power. Previous works involved with acoustic OAM relied on a PC, one or more sound-cards, or bulky electronics in order to generate the required audio signals^10,12,14^. In order to improve the affordability and compactness of the acoustic OAM generating equipment, an Arduino Due board was used in conjunction with a custom-designed filter/amplifier printed circuit board (PCB). Arbitrary frequency and phase sine-waves are generated using Direct Digital Synthesis (DDS)^34^. A block-diagram of the electronics used to produce the OAM acoustic field is shown in Fig. 6. As shown in the block-diagram, each of the 8 speakers corresponds to an audio channel, for which a signal is generated in three steps: (1) A variable duty-cycle square wave (calculated using the DDS algorithm) is produced with a PWM output of the Arduino Due board; (2) The square wave is converted into a sine wave using a low-pass Chebyshev filter built into our custom filter/amplifier board; and (3) the signal is amplified using the TDA7375V chip incorporated onto our custom filter/amplifier board, to match the optimal power range of the loudspeaker.

**Figure 7:**
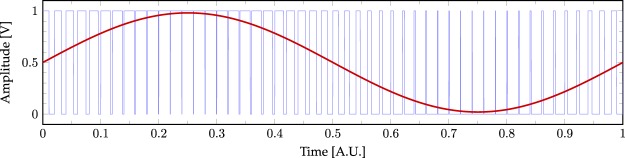
Digital synthesis of an arbitrary analogue signal. Example of a normalised sinusoidal waveform modulated using a PWM carrier wave. The duty cycle of the PWM signal (blue line) determines the amplitude of the output sinusoidal waveform (red line) after a low-pass filter operation.

In order to generate an acoustic OAM field using a phased array of speakers it is necessary to produce 8 sinusoidal signals, each out-of-phase with respect to each other. One straightforward way to generate a sinusoidal signal may be to use the on-board Digital-to-Analogue Converters (DACs) of a microcontroller. However, non-specialised microcontrollers found on hobbyist development boards such as Arduino boards, rarely have dedicated on-board DACs (even in the case of the top-range Arduino Due, only two DACs are available), meaning that it is not possible to directly synthesise more than 2 sinusoidal signals. Although it is possible to design a custom board with an arbitrary number of DAC chips, this solution is complex. Another approach to signal-generation is to use an electronic filter which is able to convert a variable duty-cycle square wave into a “smooth” signal, thus producing any arbitrary waveform within the frequency constraints of the system. Fortunately, many microcontrollers can easily produce a number of square wave signals with Pulse Width Modulation (PWM). We chose to use an Arduino Due microcontroller board, taking advantage of the ample processing power of its Atmel SAM3X8E ARM Cortex-M3 microcontroller and more importantly, its numerous PWM-output channels, allowing us to generate the desired number of phased sinusoidal-signals. The more common Arduino Uno or Nano has less built-in PWM channels but may have been usable in a non-standard manner with significant firmware development effort. We used eight PWM-output channels of the Arduino Due board to produce eight analogue sinusoidal signals, according to the following relationship:

1 $$\bar{y}=\frac{1}{T}({\int }_{0}^{D\cdot T}\,{y}_{max}dt+{\int }_{D\cdot T}^{T}\,{y}_{min}dt)=D{y}_{max}+\mathrm{(1}-D){y}_{min}$$

where *T* is the period of the carrier square-wave and *D* is its duty-cycle. The voltages that the square wave oscillates between are *y*_*min*_ and *y*_*max*_ which in this case are 0 V and 3.3 V respectively. As shown in Fig. 7 the amplitude of the output analogue signal can be engineered by changing the duty cycle of the input PWM signal with time.

**Figure 8:**
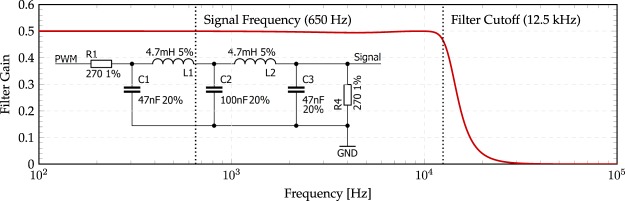
Chebyshev-filter circuit diagram and gain response. The frequency components of the input PWM signal are attenuated according to the filter gain shown. The PWM carrier frequency (328 kHz) is significantly attenuated while the signal frequency (650 Hz) is not. Resistors, inductors, and capacitors are conventionally labelled as R, L, and C respectively in the inset circuit diagram. The tolerance of the components is shown preceding a percentage sign.

The averaging operation described by Eq. 1, which is required to convert the PWM signal into an analogue waveform, is achieved electronically using a low-pass filter. The minimum PWM carrier frequency should be at least double the required analogue waveform frequency to avoid aliasing, however, the low-pass filter cutoff frequency (typically, the point where the filter gain has fallen to 0.707 of the passband gain) has to be in between these two frequencies so that it can average the square wave without adversely attenuating the desired analogue output wave. In our acoustic spanner each channel used a passive 5^*th*^ order Chebyshev filter with a cutoff of approximately 12.5 kHz, thus requiring a PWM carrier frequency at least double this. Figure 8 shows a plot of the designed filter gain as well as a circuit diagram of the filter.

Chebyshev filters have a steeper roll-off after the cutoff frequency than their simpler Butterworth counterparts (−6 dB/octave for a first order filter) at the expense of ripple, which is visible as the slight bump in gain just before the filter cutoff point. As each loudspeaker is driven with the same frequency, this ripple is not of concern and the faster rolloff improves the quality of the signal. It was found that the implementation of the Arduino *analogWrite* function contained a bug which caused signal spikes in some of the PWM channels. As a simple workaround, the PWM frequency was increased to 328 kHz, so that the spikes in the signal were almost completely suppressed by the filter. This workaround sporadically resulted in a tolerable, buzz-like distortion in the signal.

### Electronics and sound generation

High power audio amplifiers are required in order to achieve a sound pressure sufficient to impart a force on an object. Two heatsinks are visible in Fig. 5(a). These are attached to two TDA7375V, quad, 4 W Class-AB amplifiers. The amount of power that the minispeakers are able to handle for a prolonged amount of time is 1W. The extra power output of the amplifiers was required to compensate for the 50% reduction in power caused by the high-order low-pass filters, visible in Fig. 8. The resulting total harmonic distortion of the power amplifiers was estimated to be 0.03%, according to the datasheet. High audio fidelity is beneficial to the performance of the phased array and its ability to produce a pure OAM acoustic-field with the least amount of power distributed to adjacent ℓ-modes.

The DDS algorithm outputs the appropriate duty cycle values to the PWM peripherals in the microcontroller in order to generate sine waves with specific frequency and phase^34^. A *phase accumulator* variable, *P*, stores the phase, *θ*, of the wave that is to be produced using the expression sin(*θ*). The phase accumulator is an unsigned integer with a large range, in this case *n* = 32 bits which results in the range [0; 2^32^ − 1] which maps to the phases *θ* = [0; 2*π*] according to *θ* ⇒ *P* (2*π*/2^*n*^). If one modifies *θ* appropriately, with time, it is possible to produce a sine wave with arbitrary frequency and phase.

At regular intervals, dictated by a clock with frequency *f*_*c*_, a so-called *tuning word*, *M*, is added to the phase accumulator to result in the output frequency, *f*_*o*_ by the relation

2 $${f}_{o}=\frac{M{f}_{c}}{{2}^{n}}.$$

The available frequency resolution of a *n*-bit system is equal to *f*_*c*_/2^*n*^. In the case of the Arduino Due *n* = 32 bits, hence the theoretically attainable frequency resolution is quite high. However, in practice and indeed in our implementation, the mapping between the phase accumulator and the desired phase requires a truncation by division of the phase accumulator value, resulting in a reduced accuracy.

In order to make good use of the computational resources of the microcontroller, rather than calculating the value of sin(*P*(2*π*/2^*n*^)) at every update of the PWM, a precomputed lookup table of one cycle of a sine wave was stored in the memory of the Arduino Due, allowing to swiftly retrieve 256 values representing sin(*x*(2*π*/255)), where *x* ∈ [0; 255]. In this way each value of sin(*θ*) is simply retrieved using the phase accumulator, and corresponds to the value of the lookup table at index *P*/2^24^.

It is easy to add a phase shift, *ϕ*, to the output by adding to the tuning word. Since *θ* ⇒ *P*(2*π*/2^*n*^), a phase shift with respect to the original signal requires the addition of Δ*P* = 2^*n*^*ϕ*/2*π*.

### Acoustic-field maps

The acoustic field was mapped by scanning a microphone over a 12 × 12 cm^2^ area, located at ≈5 cm from the end of the 3D-printed ‘funnel’ concentrator. The y-resolution of the acoustic-maps was 0.23 mm, enabled by the fine displacement of a motorised linear stage. The x-resolution was ≈5 mm, as the x-displacement was obtained by manually translating the acoustic spanner along a rail. This was perpendicularly aligned with respect to the motorised linear-stage. The recorded signals of each scan were stitched together to produce the desired maps of the acoustic fields. In order to collect the phase-information, the signal from the scanning-microphone was recorded together with the output of one of the speakers of the acoustic spanner. In this way it was possible to retrieve the relative phase between the recorded signals at each position of the microphone. The analogue to digital conversion for the two required signals (one for the scanning-microphone and one from the reference speaker) was inexpensively realised using the left- and right-channels of a computer’s built-in sound-card. We were thus able to simultaneously record two 24-bit signals, at a sampling rate of 96,000 Hz, taking advantage of the impressive processing ability of widely-available, consumer-grade sound-cards.

## Data Availability

The design files of the 3D-printed spanner and schematics of the custom filter/amplifier PCB together with the signal-generating code required to program the Arduino Due microcontroller board have been deposited in Enlighten with the identifier, 10.5525/gla.researchdata.628 and can also be found at https://github.com/witseie/AcousticSpanner.
